# Does physical therapy prevent post-operative delay in return of function following tension-band plating?

**DOI:** 10.1007/s11832-015-0700-x

**Published:** 2015-10-26

**Authors:** Yale A. Fillingham, Tyler Luthringer, Brandon J. Erickson, Monica Kogan

**Affiliations:** Department of Orthopaedic Surgery, Rush University Medical Center, 1611 West Harrison Street, Chicago, IL 60612 USA

**Keywords:** Guided growth, Tension-band plate, Angular deformity, Physical therapy, Pediatric knee

## Abstract

**Purpose:**

The clinical outcomes and complications of tension-band plating have been well documented, and commonly include a post-operative delay in return of function. We performed a retrospective comparison study to evaluate the capacity of immediate post-operative physical therapy to prevent this post-operative delay in return of function.

**Methods:**

Sixty-seven consecutive growth-deformity patients who were treated with tension-band plating at a single institution fulfilled the study criteria. Patients were allocated into two treatment groups: no post-operative physical therapy and immediately post-operative physical therapy. All patients were evaluated for delayed return of function, which was defined as use of crutches, lack of >90° flexion and full extension of the knee, or persistent pain requiring medication at the initial 2-week follow-up visit. Rates of delayed function were compared between the two treatment groups.

**Results:**

Among the study participants, 48 patients had no physical therapy and 19 patients had immediate post-operative physical therapy. Eighteen patients in the no physical therapy group reported a delay (37.5 %) while only 2 patients in the physical therapy treatment group reported a delay (10.5 %); *p* = 0.0386.

**Conclusions:**

Delayed return of function dramatically affects pediatric patients, causing unnecessary absence from school and strain on the caregiver. Therefore, it is important to identify treatment modalities to help mitigate the complications of surgery. We conclude that the use of immediate post-operative physical therapy statistically significantly helps patients to return more rapidly to their functional level.

## Introduction

Growth deformities are among the most common consultations for a pediatric orthopedist [1]. Historically, the surgical treatment options included osteotomies or permanent epiphysiodesis; however, the concept of temporary epiphysiodesis brought forth new methods for managing growth deformities [2–5]. The techniques of temporary epiphysiodesis described in the literature include Blount staples, transphyseal screws, and tension-band plating. In response to the pitfalls of Blount staples, Dr. Peter Stevens developed the tension-band plate, consisting of two screws and a two-hole plate [6]. The tension-band design helps eliminate the compressive force of the staple across the growth plate and provides rigid screws to help resist implant extrusion [1, 7, 8].

Until recently, current literature on complications of the tension-band plate had focused on re-operation following rebound growth and plate breakage in the setting of Blount’s disease [7, 9, 10]. In a recent case series, Fillingham et al. highlighted a more common complication—delayed return of function—in 37 % of patients at 2 weeks after the use of the tension-band plate [11]. The study defined a delay in return of function as continued use of crutches, diminished range of motion (ROM), or persistent pain. Patients 11 years of age and older, patients with four or more plates, or patients with femoral plates were most at risk of delayed function. As a result of the study by Fillingham et al., our clinical practice changed to routinely include immediate post-operative physical therapy (PT) with the anticipation of preventing symptoms of delayed return of function [11].

We present a retrospective comparative study to investigate the effectiveness of immediate post-operative PT in limiting the incidence of delayed return of function following tension-band plating.

## Methods and patients

### Study design and setting

We retrospectively compared a series of 67 patients with a growth deformity who underwent guided growth using a tension-band plating system, which included the Eight-Plate^®^ (Orthofix, McKinney, TX, USA) and PediPlate^®^ (OrthoPediatrics, Warsaw, IN, USA) from October 1, 2006 to January 30, 2015. Patients were separated into two treatment groups: no post-operative PT and post-operative PT. The study was conducted under the approval of the institutional review board at our institution.

### Study subjects

Indications for surgery in the study were patients with open growth plates along with a diagnosis of limb length inequality, angular deformity, or combination of limb length inequality and angular deformity. The only contraindication to surgery was patients with closed growth plates. The only exclusion criteria for the study were patients with less than 2 weeks of post-operative follow-up. Based on the inclusion and exclusion criteria, a consecutive series of 67 patients were identified for the study. There were 43 male patients and 24 female patients. Their average age was 11 years (range 2–18 years). Diagnoses included limb length inequality (32), genu valgum (25), Blount’s disease (6), and four other diagnoses, each relating to only one patient. Number of plates implanted per patient included one (27), two (28), three (2), four (8), five (1), and six (1). All plates were implanted on the lower extremity that included the distal femur only (30), the distal femur and proximal tibia (22), and the proximal tibia only (15).

### Surgery and post-operative care

All patients were treated by the senior surgeon. The procedure was performed following the manufacturer’s operative technique. Postoperatively, all patients were provided with crutches strictly for comfort measures, but were instructed to be full weight-bearing. Regardless of whether or not the patient received immediate post-operative PT, all were encouraged to return to normal activity as tolerated on the affected lower extremity and to discontinue the crutches as soon as was tolerable.

After discharge from the hospital, all on an outpatient basis, the patients were followed in the clinic at 2 weeks and then at intervals of 3–6 months to document progression and identify the timing of hardware removal. Each routine follow-up in the clinic included assessments of strength, range of motion, functional status, and a radiographic investigation to evaluate the correction of the growth deformity and the hardware position.

### Outcome measures

We retrospectively reviewed the defined patient population, which included verifying the diagnosis, operative date, locations of plates implanted, and number of plates implanted. Patients were investigated for the presence of a post-operative delay in return of function at the initial 2-week follow-up appointment, which was defined as use of crutches, knee ROM of less than full extension, and flexion of <90°, or persistent pain requiring medication.

### Statistical analysis

Patient demographics were evaluated for dissimilarities between the treatment groups. Analysis was done using a two-tailed Student’s *t*-test for continuous variables, and a chi-square or Fisher’s exact test was used for categorical variables. Comparison of the incidence of a post-operative delay in return of function between the two treatment groups was done with a two-tailed Fisher’s exact test. For all tests performed within the study, statistical significance was defined as a *p* value of less than 0.05.

## Results

Among the 67 study participants, 48 patients had no PT and 19 patients had immediate post-operative PT. The average age in the treatment groups of no PT and immediate post-operative PT was 10.8 years (range 2–18 years) and 11.5 years (range 4–15 years), respectively. One (20 patients), two (21), three (2), four (4), or five (1) plates were implanted in the no-PT patients. The locations of the plates in the no-PT patients included the femur only (22), the femur and tibia (16), and the tibia only (10). One (7 patients), two (7), four (4), or six (1) plates were implanted in the patients who received post-operative PT. The locations of the plates in the patients who received post-operative PT included the femur only (8), the femur and tibia (6), and the tibia only (5). None of the measurable characteristics showed a statistically significant difference between the two treatment groups (Table 1).

**Table 1:** Patient characteristics of the treatment groups

	No PT (*n* = 48)	PT (*n* = 19)	*P* value
Age (years)	10.8	11.5	0.41
Sex			0.16
Female	20	4	
Male	28	15	
Number of plates implanted			0.33
1	20	7	
2	21	7	
3	2	0	
4	4	4	
5	1	0	
6	0	1	
Location(s) of plates			0.89
Femur only	22	8	
Femur/tibia	16	6	
Tibia only	10	5	

In total, 20 of the 67 patients (30 %) experienced a delay in return of function. Among the 18 patients in the no-PT group who experienced a delay of function, nine experienced diminished ROM, three were still using crutches, three had persistent pain, and three continued to have pain and diminished ROM. Only two patients of the immediate post-operative PT group demonstrated a delay, with both patients experiencing persistent pain and diminished ROM. Overall, eighteen patients in the no-PT group had reported a delay (37.5 %) while only two patients in the PT treatment group had reported a delay (10.5 %); *p* = 0.0386.

## Discussion

Tension-band plating has become a modality of choice in the treatment of growth deformities, secondary to its high rates of successful correction with rare complications [6, 10, 12–18]. While the consensus in post-operative management has been to encourage early weight-bearing, range of motion, and return to activity after tension-band plating, the existing literature lacks any evaluation of the utility of early postoperative PT in promoting such a return of function [10, 12, 13, 17]. In a previous study, Fillingham et al. identified risk factors associated with a delay in return of function following tension-band plating [11]. In that investigation, 92.3 % of the patients with delayed return of function experienced complete resolution of symptoms after PT [11]. In the current study’s analysis, we validated the efficacy of preemptive PT in preventing a delay in return of function during the immediate postoperative period after the use of the tension-band plates. Our findings conclude that subjects who undergo immediate PT are able to more rapidly return to their preoperative functional status and activity level than those who do not. We believe the conclusions of this study should prompt providers to preemptively prescribe postoperative PT to limit the negative social implications associated with a delay in return of function for patients and caregivers alike.

A number of limitations to this study should be acknowledged. First, our relatively small, heterogeneous sample size precludes evaluation of which preoperative diagnoses and degrees of correction benefit most from immediate postoperative PT. In the presented study, five of 19 patients who underwent PT in the immediate postoperative period had four or more plates placed while six of 19 patients underwent bilateral operations; five subjects met both of these criteria. Since neither of the two subjects who experienced a delay in return of function despite undergoing immediate PT had more than four plates or bilateral procedures, we do not believe our observations to be biased by the predominance of less extensive procedures performed within the sample. Second, the postoperative PT protocols were not standardized across the patient sample. As the patients in the study were allowed to participate in PT at a variety of recommended centers with a number of pediatric physical therapists, we cannot make claims as to which elements of the rehabilitation protocols proved most beneficial. Third, there was no emphasis on long-term follow-up in the study. We believe this does little to detract from our conclusions, as the focus of the investigation was on a return to function within the immediate post-operative period. Fourth, patients with persistent pain did not have an assigned visual analog scale for pain, so any level of pain requiring continued medication defined persistent pain. However, we believe the definition of pain used describes the criterion in accordance with the outcome of interest. Lastly, limitations are inherently present secondary to the retrospective study design.

Discussion in the literature of the immediate post-operative course and timing of return to normal activity after tension-band plating is limited. In a prospective series of 34 consecutive patients with 65 angular deformities who underwent a guided growth procedure, Stevens et al. required no immobilization or restrictions on early ambulation, encouraging a return to activity as tolerated [17]. While physical therapy was eventually prescribed for “a minority of patients” who were slow or apprehensive to mobilize, the frequency of occurrence as well as the success of delayed PT was not reported. Similarly, in an evaluation of the efficacy of the tension-band plate for angular deformity correction in children under 5 years of age, Burghardt et al. failed to report details of the outcomes of PT prescribed for patients who experienced reduced ROM of the knee at the initial postoperative visit (1–2 weeks after surgery) [13]. Although experiences of postoperative delay in return of function after guided growth procedures have been reported, there has been little effort to investigate how best to prevent such delays in function until the presented study [11, 13, 17]. Using a two-tailed Fisher’s exact test, we have demonstrated the statistical significance of immediate postoperative PT in preventing a delay in return of function (*p* = 0.0386).

Fillingham et al. have previously identified an increased rate of delayed post-operative return of function with the use of a greater number of plates, bilateral operations, and use of femoral plates for the correction of lower limb angular deformities and limb length discrepancy [11]. Regarding the etiology of the delayed function with the use of more of plates and bilateral operations, it is intuitive that both lead to more soft tissue manipulation, contributing to increased post-operative pain and range of motion impairment. In the presented study, five of 19 and six of 19 patients who underwent PT in the immediate postoperative period underwent placement of four or more plates and bilateral operations, respectively, with five subjects meeting both of these criteria. Following the use of immediate postoperative PT, however, none of these patients experienced a delay in return of function secondary to pain, reliance on crutches, or reduced ROM, attesting to the benefit of PT even after a more intensive operative intervention.

In Fillingham et al., the authors observe that 18 of 19 patients who received at least one distal femur plate experienced a post-operative delay in function [11]. Consistent with these findings, Burghardt et al. note that PT is more likely to be needed after femoral than tibial plates because of limited ROM of the knee [13]. In our current evaluation of the capacity of postoperative PT to prevent delayed return of function, the only two patients who experienced decreased function despite PT underwent distal femoral manipulation. Fillingham et al. discuss the surgical approach to the distal femur and hypothesize why femoral plates tend to be more associated with functional pain, use of crutches, or impaired range of motion [11]. In a recent cadaveric study, Bachmann et al. performed temporary hemiepiphysiodesis with a plate and two screws at the distal medial femur in eight adult specimens [19]. Following surgical manipulation, they dissected specimens to assess soft and connective tissue damage. Based on their findings, they concluded that correct plate placement for temporary hemiepiphysiodesis of the distal medial femur frequently leads to damage to the medial patellofemoral ligament (MPFL) (six of eight specimens), soft tissue impingement, and accidental arthrotomy of the knee joint (four of eight specimens) [19]. During normal knee motion, medial capsular structures including the MPFL glide over the surface of the medial femoral condyle; we would therefore expect impingement of the anterior aspect of the MPFL or joint capsule by a plate to hinder motion and cause pain during knee flexion.

Although Fillingham et al. demonstrated that patients 11 years of age or older were at statistically significantly greater risk for a delay in return of function, we believe their finding to be an artifact of other factors not explicitly related to an underlying physiological process unique to older patients [11]. When older patients present with conditions indicated for treatment with guided growth, a more aggressive treatment plan is required due to the limited period of time to obtain the desired correction. Consistent with this notion, the group of patients 11 years of age and older in the study by Fillingham et al. consistently showed a higher incidence of using more than one plate, requiring bilateral operations, or using femoral plates [11]. In the current study’s analysis, while the two patients who experienced a delay in return of function despite postoperative PT were aged 13 and 15 years, neither patient received more than two plates or underwent bilateral procedures. This re-introduces the possibility that older patients may be at individual risk for a delay in return of function despite the use of PT and irrespective of the complexity of the procedure undertaken.

As tension-band plating continues to become more widely used in the treatment of growth deformities, we need to not only be able to identify the patients at risk for a delayed return of function, but to also be able to implement prophylactic management to minimize such a delay where appropriate. Issues relating to pain, limitations on range of motion, and reliance on crutches during the postoperative period are associated with the inability to return to school in a timely manner, which has important social implications for patients and caregivers alike. By minimizing the occurrence of such issues, we can allow patients and family members to more readily resume their basic level of function after surgery. To our knowledge, the presented study is the first of its kind to investigate the effectiveness of preemptive PT in preventing a delay in return of function during the immediate postoperative period after the use of tension-band plating for correction of growth deformity. Based on our study, we conclude that immediate postoperative PT statistically significantly prevents delays in return of function. We recommend the implementation of rehabilitation protocols to help avoid delays in the return to the preoperative level of function.
